# Urban residents’ health literacy of four major cancers: a cross-sectional national survey

**DOI:** 10.3389/fpubh.2026.1762797

**Published:** 2026-06-17

**Authors:** Yang Lyu, Wei Guo, Zhen Wang, Catherine Paterson, Frances Lin

**Affiliations:** 1 Department of Thoracic Surgery, National Cancer Center/National Clinical Research Center for Cancer/Cancer Hospital, Chinese Academy of Medical Sciences and Peking Union Medical College, Beijing, China; 2 College of Nursing and Health Sciences, Flinders University, Bedford Park, Adelaide, SA, Australia; 3 Caring Futures Institute, Flinders University, Bedford Park, Adelaide, SA, Australia

**Keywords:** cancer, China, health literacy, survey, urban residents

## Abstract

**Background:**

Cancer poses a significant global healthcare challenge, and individual health literacy directly impacts screening and the adoption of self-care behaviors.

**Objectives:**

This study aimed to compare health literacy scores for four cancers (colorectal, esophageal, lung, and kidney) among Chinese urban residents and to identify associated factors.

**Methods:**

A descriptive cross-sectional study recruited 1,226 urban adults via professional networks across China. Participants provided demographic data and completed a validated health literacy questionnaire. Multiple linear regression was used to analyze associations with health literacy scores.

**Results:**

Health literacy scores ranked as follows: colorectal (7.50 ± 3.35), esophageal (6.27 ± 3.15), lung (5.88 ± 3.07), and kidney (4.30 ± 2.89). Education and household income emerged as consistent predictors of health literacy across all cancers. Age, sex, employment status, and a family history of cancer were associated with colorectal cancer health literacy scores. Participants who reported higher levels of health literacy scores in esophageal cancer were older and had a previous cancer diagnosis. Participants who reported higher lung cancer health literacy scores were older, female, retired, and had a family history of cancer. Factors contributing to higher kidney cancer health literacy scores included education, annual household income, and non-smoking status.

**Conclusion:**

Urban residents demonstrated moderate levels of cancer-related health literacy, and public health strategies should be targeted toward people with lower education and reduced annual household income. Implication for Practice: This research highlights the need for targeted educational interventions in community healthcare settings to identify urban residents at high risk of low health literacy, ultimately strengthening the reach and impact of cancer screening and prevention programs.

## 1 Introduction

Cancer is one of the major challenges faced by healthcare systems worldwide (1). According to the World Health Organization, by 2040, cancer incidence is projected to double compared with that recorded in 2018 (18.1 million), and 20% of the global population is expected to develop cancer in their lifetime (2). Globally, the burden of cancer has increased and continues to be a major public health issue with significant morbidity and mortality (3). Cancer is currently ranked as the second leading cause of non-communicable disease-associated mortality in China (4). In 2022, there were 4,824,700 new cancer cases and 2,574,200 deaths in China (4). The higher cancer prevalence has been attributed to the expanding aging population in China over recent decades. Moreover, this trend is also associated with modifiable cancer-related risk factors, such as tobacco use (and passive smoking), obesity, poor diet, and physical inactivity (4). For example, China’s smoking rate for individuals aged 15 years and above was 26.6% in 2018, with a staggering 68.1% of non-smokers exposed to second-hand smoke in public areas (5), which was attributed to 44.7% of male lung cancer deaths and 6.4% of female lung cancer deaths (5). To address modifiable cancer-related risk factors, global efforts have developed policies and national cancer care programs, including those in China, to improve public health literacy and awareness and to promote engagement in health-promotion behaviors (6).

Cancer health literacy refers to an individual’s ability to obtain, process, and understand health information and services needed to make informed health decisions (7), which is essential for people’s awareness and knowledge of cancer-related health services, adherence to screening, and preventive measures that will help to reduce the incidence and prevalence of cancer (7, 8). Some studies (9–11) have identified that patients with limited cancer health literacy suffer from poor treatment compliance and reduced participation in healthcare decision-making and present with an advanced stage of cancer at diagnosis. Evidence has underscored that cancer health literacy prevention knowledge could prevent more than 40% of cancer deaths (12). Cancer health literacy interventions through public awareness and educational programs are essential to optimize the quantity and quality of life (13).

Health literacy influences cancer outcomes through multiple pathways. Individuals with adequate health literacy are more likely to recognize warning signs, understand screening recommendations, adhere to follow-up protocols, and communicate effectively with healthcare providers. Conversely, limited health literacy has been associated with delayed presentation, lower screening uptake, and poorer treatment adherence (14, 15).

There have been various efforts to enable cancer health promotion and prevention programs to raise awareness of cancer, particularly related to its risk factors, and the importance of screening to enable timely and early diagnosis (16) against the persistent negative attitudes and stereotypes toward cancer. Arguably, cancer health literacy could have an important role in mitigating the cancer burden; however, there is no evaluation of cancer health literacy. The following main gaps have been identified in relation to cancer control in China. First, most published studies to date have been limited to measuring awareness of health promotion and prevention knowledge among general cancer (17) or one type of specific cancer, for example, breast cancer (18) or colorectal cancer (19), and are limited to knowledge of target risk factors with very small sample sizes, limiting generalizability. Second, the existing studies did not use standardized instruments with demonstrated reliability and validity (17). Consequently, this methodological shortcoming further limits comparability among study results and further limits existing status of the levels of health literacy among the Chinese population about cancer prevention and control.

We acknowledge that several large-scale studies have investigated cancer awareness using publicly available databases (20, 21). However, these studies often relied on single-item awareness questions rather than validated multi-item scales, and few have simultaneously compared health literacy across multiple cancer types using a standardized instrument. Our study addresses these gaps by developing and applying a validated, cancer-specific health literacy scale to enable direct comparisons across four major cancers.

The selection of colorectal, esophageal, lung, and kidney cancers for this study was based on three considerations. First, epidemiological data show that colorectal, esophageal, and lung cancers rank among the top five in both incidence and mortality in China (4). Second, these four cancers share strong associations with modifiable lifestyle factors (e.g., diet, smoking status, and alcohol consumption), making them particularly amenable to prevention through improved health literacy. Third, lung and kidney cancers are often termed “silent cancers” due to their asymptomatic early stages, presenting unique challenges for early detection that warrant targeted literacy assessment. While kidney cancer has a lower incidence than the other three, its rapidly increasing trend and particularly low public awareness make it a critical target for health literacy research.

To the best of our knowledge, no study has evaluated the public’s level of health literacy across multiple types of cancer. Thus, the current study aimed to develop a series of cancer health literacy scales and then investigate and compare the health literacy of major cancers among urban residents. The findings from this study aimed to inform the development and implementation of targeted health promotion and prevention strategies and policies.

## 2 Methods

### 2.1 Study design and sample

This study is a cross-sectional descriptive survey following the Strengthening the Reporting of Observational Studies in Epidemiology (STROBE) Statement (22). A convenience and snowball sampling recruitment strategy was used in this study to reach our study population across China, to attain breadth of understanding. The study population was urban residents in China who were (1) older than 18 years old; (2) able to complete the questionnaire in Chinese; and (3) able to provide informed written consent.

This study focused on urban residents because (1) urban populations have better Internet access, facilitating online survey distribution; (2) cancer screening programs are more established in urban areas, making health literacy assessment particularly relevant; and (3) rural populations may face different barriers (e.g., lower education and limited healthcare access) that warrant separate investigation. We acknowledge this as a limitation, and future studies should specifically target rural populations.

### 2.2 Instruments and measurements

The questionnaire contained two parts:

1. Sociodemographic characteristics of the residents: Sociodemographic characteristics included age, sex, living status, marital status, educational background, employment status, annual household income (RMB/year) in 2022, existing co-morbidities (including previous diagnosis of cancer), family history of cancer, and smoking and alcohol behaviors.
2. Cancer health literacy scale in Chinese: This scale is adapted for the four cancers, namely colorectal, esophageal, lung, and kidney cancers. Each cancer scale contained 10 single-choice questions that reflected items of cancer prevention beliefs, lifestyle practice, early screening, warning signs and symptoms, and examination of cancer, following the guidelines of prevention and early screening for cancer (18, 19). In total, each cancer section was based on three considerations, namely early signs and symptoms recognition (2 items), preventing perception and strategies (5 items), and early screening and seeking medical help (3 items). The response options were “true,” “false,” or “do not know.” Correct response was scored as 1, while incorrect or “do not know” response was scored as 0. The total score was calculated for all 10 questions, which ranged from 0 to 10, with higher scores indicating a higher level of health literacy of cancers.

### 2.3 Reliability and validity of the cancer health literacy scale

Although several validated health literacy instruments exist, none were specifically designed to assess literacy across multiple types of cancer (colorectal, esophageal, lung, and kidney) simultaneously using a standardized format. Therefore, we developed a new scale.

Ten purposely selected Chinese experts, including two biostatisticians, two oncology nursing specialists, two cancer prevention specialists, and four cancer care medical specialists in colorectal, esophageal, lung, and kidney cancers, were invited to review the instrument to inform the reliability and validity of the scales. The content validity value for all scales was 1.0, indicating good content validity (23). After establishing content validity, a pilot study was conducted from 25th to 28th February 2025 with 130 urban residents from two cities to test the reliability of the survey. These 130 urban residents were not included in the final electronic survey. The internal consistency reliability of the Cancer Health Literacy Scale was good (23), with Cronbach’s alpha values of 0.870 (colorectal cancer), 0.892 (esophageal cancer), 0.905 (lung cancer), and 0.9517 (kidney cancer), respectively. The test–retest coefficients (2 weeks apart) were also high (23), with values of 0.886, 0.887, 0.905, and 0.910 for colorectal cancer, esophageal cancer, lung cancer, and kidney cancer, respectively. In addition, principal component analysis (PCA) was performed to test the structural validity. Promax rotation and Kaiser’s criteria (eigenvalue > 1) were used for factor extraction. Bartlett’s sphericity test indicated that the sample was adequate for factor analysis of the four scales.

### 2.4 Data collection

Data were collected from 1 March 2025 to 1 September 2025. The Cancer Health Literacy Scale survey was disseminated via a Chinese electronic platform (Qing Song Chou). A unique link was sent to 10 liaison staff from the Chinese Cancer Love Fund via WeChat and then sent to the WeChat group for their communities. The survey contained a participant information section, which detailed the purpose and significance of this study. Residents who agreed to participate were required to click the ‘Agree’ button to start the survey, indicating their informed consent. If individuals did not click the “Agree” button, they were not able to access the online survey. Each WeChat account could only submit one survey to avoid duplicate submissions. The survey took approximately 15–20 min to complete. Question logic was used to support the quality of participants’ responses. For example, the answer for age was required to be a number ranging from 18 to 99. Additionally, to avoid missing data, each question was designed to require an answer.

### 2.5 Statistical analysis

At the end of the survey period, electronically collated questionnaires were entered into SPSS version 25 (IBM Corporation, Armonk, NY, USA), checked for completeness, and cleaned in preparation for analysis. Normality of continuous variables was assessed using the Kolmogorov–Smirnov test and visual inspection of Q-Q plots and histograms. The health literacy scores for all four cancers were approximately normally distributed (Kolmogorov–Smirnov test *p* > 0.05 for all four scales). Continuous data were therefore described as means and standard deviations (SD). Categorical variables were described using frequencies and percentages. To explore the association between the health literacy scores and demographic characteristics of the sample, independent sample *t*-tests, one-way analysis of variance with Levene’s test to assess homogeneity of variance, and Pearson’s correlation and non-parametric test were utilized depending on the variable and distribution of data. Moreover, multiple linear regression was used to analyze the association between demographic characteristics and the health literacy scores. The final model estimated the mean change in cancer health literacy scores and standardized regression coefficient (*β*) for the linear trend for a given independent variable (age, sex, living status, marital status, educational background, employment status, annual household income (RMB/year), existing co-morbidities (including previous diagnosis of cancer), family history of cancer, and smoking and alcohol behaviors) as well as the 95% confidence intervals (CIs). The alpha values were two-tailed. The *p-*value for statistical significance was set at 0.05.

Before conducting multivariable linear regression, we performed several sensitivity analyses. Multicollinearity was assessed using the variance inflation factor (VIF), with all VIF values < 2.5, indicating no significant collinearity among independent variables. Residual plots were examined to verify homoscedasticity and linearity assumptions. The outliers were identified using studentized residuals (>3), and no influential outliers were detected. These analyses confirmed the appropriateness of the multiple linear regression models.

### 2.6 Ethical consideration

The Ethics Committees of the National Cancer Center approved the study (approval no. 25/016–5,741). The online survey was anonymous, and following a review of the study information, the completion and return of the questionnaire indicated consent.

## 3 Results

### 3.1 Sample characteristics

A total of 1,226 respondents completed and returned the survey. The average age of respondents was 42.04 ± 14.84 years (ranging from 18 to 83), with 54.2% (*n* = 664) being female. Of the respondents, 28.4% (*n* = 348) had existing co-morbidities, 19.7% (*n* = 242) had a family history of cancer, and 3.0% (*n* = 37) were diagnosed with cancer. Of the respondents, 23.2% (*n* = 284) were identified as current smokers, and 33.6% (*n* = 412) consumed alcohol regularly. The detailed demographic characteristics of the respondents are presented in Supplementary Table 1.

To assess whether the inclusion of participants with a cancer diagnosis (*n* = 37, 3.0%) biased the results, we conducted a sensitivity analysis by excluding these participants and re-running the multivariable regression models. The pattern of results remained unchanged (data not shown), suggesting that the inclusion of cancer-diagnosed individuals did not materially affect our conclusions.

### 3.2 Comparison of health literacy of four cancers

**Figure 1:**
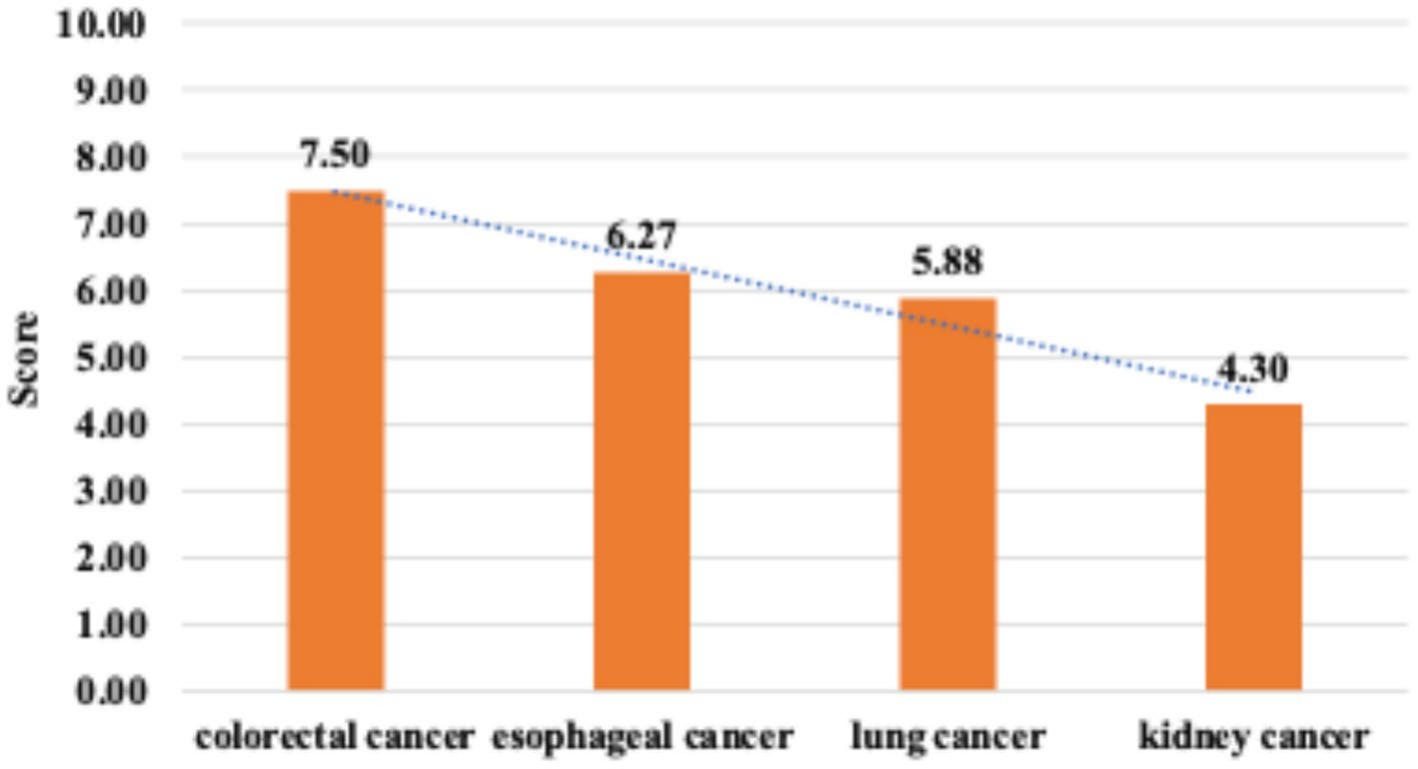
Health literacy of four cancers (*n* = 1,226).

**Figure 2:**
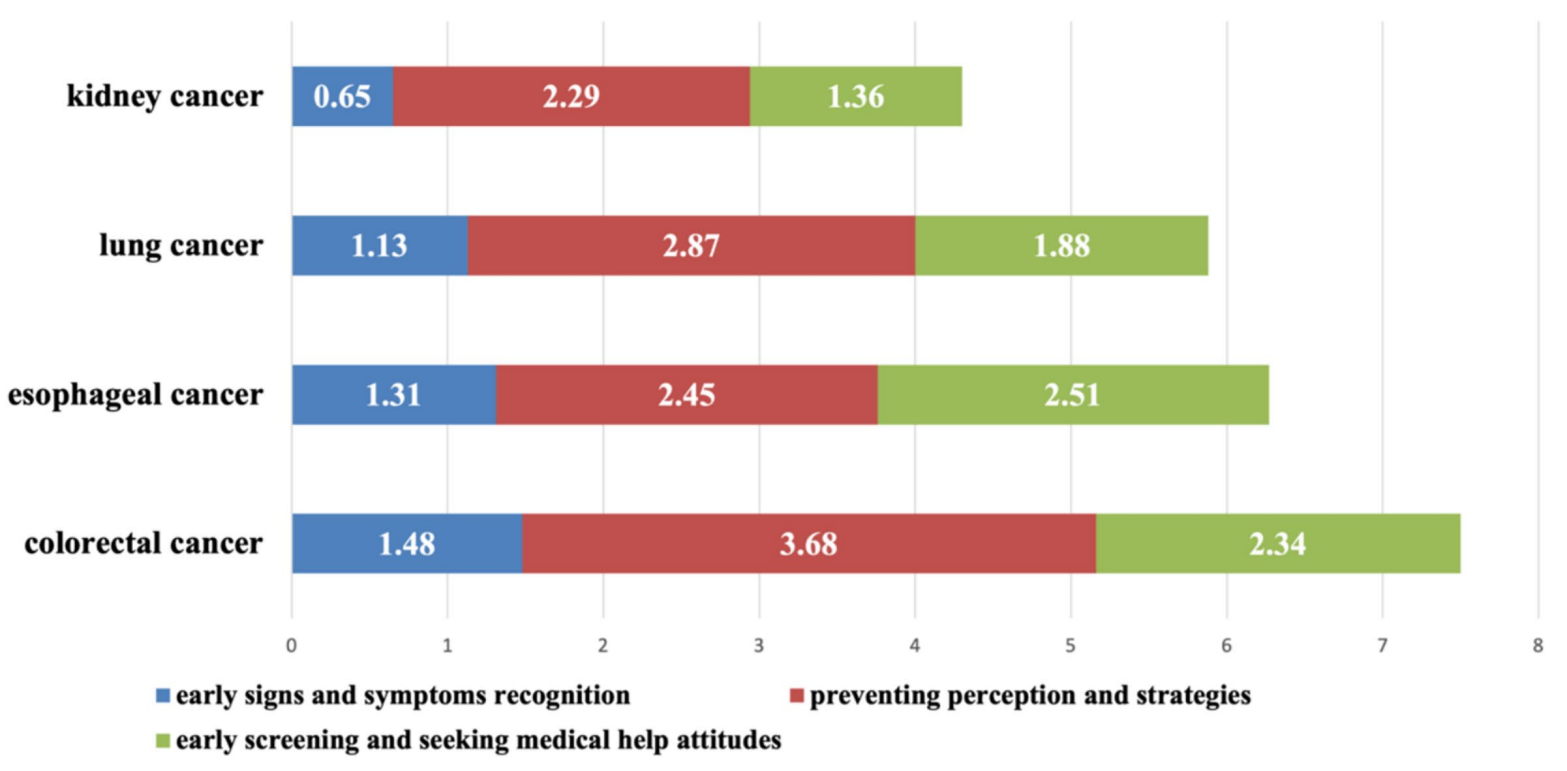
Three aspects of health literacy of four cancers (*n* = 1,226).

The ranking of the health literacy of four cancers was as follows: colorectal cancer (7.50/10 ± 3.354, ranging from 2 to 10), esophageal cancer (6.27/10 ± 3.150, ranging from 1 to 10), lung cancer (5.88/10 ± 3.068, ranging from 1 to 10), and kidney cancer (4.30/10 ± 2.891, ranging from 0 to 10) (See Figure 1). Figure 2 presents the three aspects of the total health literacy of four cancers, in which kidney cancer health literacy exhibited consistently lower scores in all three aspects.

### 3.3 Details of health literacy of four cancers

The detailed results of health literacy of four cancers are demonstrated in Tables 1–4. In terms of colorectal cancer, the majority (*n* = 1,007, 82.1%) of respondents perceived that “patients with bloody stools or alternating diarrhea and constipation should see a doctor”, while only 66.5% (*n* = 815) of the respondents perceived “drinking less alcohol could reduce the risk of colorectal cancer”. In terms of health literacy of esophageal cancer, most respondents (*n* = 1,031, 84.1%) perceived that “people whose family member has esophageal cancer should actively participate in early screening”, yet only half of the respondents (*n* = 663, 54.1%) recognized that “early esophageal cancer can have sternal distension or slight pain”. In addition, for lung cancer-related health literacy, nearly 80% (n = 979) of the respondents knew people with a history of lung disease should be screened regularly, while less than half (*n* = 523, 42.7%) knew that an increasing number of women who never smoked could get lung cancer. Moreover, in terms of kidney cancer-related health literacy, 67.9% (*n* = 832) knew that diets high in fat, protein, oil, and salt increase their risk of kidney cancer, while only 20.6% (*n* = 252) of the respondents knew how to detect kidney cancer early.

**Table 1:** Respondents’ health literacy of colorectal cancer (*n* = 1,226).

Items	Correct rate	True	False	Do not know
Patients with bloody stools or alternating diarrhea and constipation should see a doctor	1,007 (82.1%)	1,007 (82.1%)	41 (3.3%)	178 (14.5%)
Relatives with colorectal cancer should actively participate in the census	988 (80.6%)	988 (80.6%)	48 (3.9%)	190 (15.5%)
Excessive consumption of grilled or fried smoked meats can increase the risk of colorectal cancer	956 (78.0%)	956 (78.0%)	39 (3.2%)	231 (18.8%)
Developing good bowel habits can prevent colorectal cancer	951 (77.6%)	951 (77.6%)	56 (4.6%)	219 (17.9%)
Reasonable and high-fiber diet can prevent colorectal cancer	936 (76.3%)	936 (76.3%)	49 (4.0%)	241 (19.7%)
Fresh fruits and vegetables regularly can prevent colorectal cancer	928 (75.7%)	928 (75.7%)	47 (3.8%)	251 (20.5%)
Screen can detect colorectal cancer in its early stages	913 (74.5%)	913 (74.5%)	59 (4.8%)	254 (20.7%)
Maintaining a proper weight and exercising can prevent colorectal cancer	883 (72.0%)	883 (72.0%)	62 (5.1%)	281 (22.9%)
Colonoscopy is an effective early screening method for colorectal cancer	817 (66.6%)	817 (66.6%)	75 (6.1%)	334 (27.2%)
Drinking less alcohol could reduce the risk of colorectal cancer	815 (66.5%)	815 (66.5%)	81 (6.6%)	330 (26.9%)

Data are presented as *n* (%).“Correct rate” indicates the number and percentage of respondents who selected the correct answer (either “true” or “false” depending on the item).

**Table 2:** Respondents’ health literacy of esophageal cancer (*n* = 1,226).

Items	Correct rate	True	False	Do not know
People whose family member has esophageal cancer should actively participate in early screening	1,031 (84.1%)	1,031 (84.1%)	45 (3.7%)	150 (12.2%)
Eating fresh vegetables, meat, eggs, milk, and other foods regularly can prevent esophageal cancer	865 (70.6%)	865 (70.6%)	72 (5.9%)	289 (23.6%)
Early symptoms of esophageal cancer are discomfort in swallowing or a slight choking sensation	826 (67.4%)	826 (67.4%)	56 (4.6%)	344 (28.1%)
Eat hot food frequently is not associated with the incidence of esophageal cancer	795 (64.8%)	215 (17.5%)	795 (64.8%)	216 (17.6%)
Gastroscopy is an effective early screening method for esophageal cancer	721 (58.8%)	721 (58.8%)	86 (7.0%)	419 (34.2%)
Frequent eating overnight meals/preserved foods is not associated with esophageal cancer	712 (58.1%)	243 (19.8%)	712 (58.1%)	271 (22.1%)
Alcohol consumption is not associated with the incidence of esophageal cancer	706 (57.6%)	163 (13.3%)	706 (57.6%)	356 (29.0%)
Smoking is not associated with the incidence of esophageal cancer	678 (55.3%)	195 (15.9%)	678 (55.3%)	353 (28.8%)
Imaging examination (B ultrasound, CT, etc.) is the most accurate way to diagnose esophageal cancer	664 (54.2%)	664 (54.2%)	135 (11.0%)	427 (34.8%)
Early esophageal cancer can have sternal distension or slight pain	663 (54.1%)	663 (54.1%)	56 (4.6%)	507 (41.4%)

Data are presented as *n* (%). “Correct rate” indicates the number and percentage of respondents who selected the correct answer (either “true” or “false” depending on the item).

**Table 3:** Respondents’ health literacy of lung cancer (*n* = 1,226).

Items	Correct rate	True	False	Do not know
People with a history of lung disease (such as tuberculosis, chronic obstructive pulmonary disease, and pulmonary fibrosis) should be screened regularly	979 (79.9%)	979 (79.9%)	50 (4.1%)	197 (16.1%)
Annual CT physical examination is necessary for people at high risk of lung cancer	957 (78.1%)	957 (78.1%)	45 (3.7%)	224 (18.3%)
You cannot get lung cancer if you do not smoke	923 (75.3%)	75 (6.1%)	923 (75.3%)	228 (18.6%)
Long-term cooking and exposure to cooking fumes increases the risk of lung cancer	868 (70.8%)	868 (70.8%)	59 (4.8%)	299 (24.4%)
Low-dose chest CT is the most appropriate method for early detection of lung cancer	669 (54.6%)	669 (54.6%)	89 (7.3%)	468 (38.2%)
Lung cancer is the cancer with the highest death rate in China	622 (50.7%)	622 (50.7%)	131 (10.7%)	473 (38.6%)
Irritant dry cough is the main clinical symptom of early lung cancer	572 (46.7%)	572 (46.7%)	120 (9.8%)	534 (43.6%)
Lung CT cannot determine the pathological type of lung cancer	542 (44.2%)	542 (44.2%)	168 (13.7%)	516 (42.1%)
Lung cancer may be asymptomatic in its early stages	536 (43.7%)	536 (43.7%)	228 (18.6%)	462 (37.7%)
More and more women who never smoke get lung cancer	523 (42.7%)	523 (42.7%)	223 (18.2%)	480 (39.2%)

Data are presented as *n* (%). “Correct rate” indicates the number and percentage of respondents who selected the correct answer (either “true” or “false” depending on the item).

**Table 4:** Respondents’ health literacy of kidney cancer (*n* = 1,226).

Items	Correct rate	True	False	Do not know
Diets high in fat, protein, oil, and salt increase the risk of kidney cancer	832 (67.9%)	832 (67.9%)	70 (5.7%)	324 (26.4%)
Early screening for kidney cancer is abdominal B-scan	612 (49.9%)	612 (49.9%)	90 (7.3%)	523 (42.7%)
Family history of kidney cancer does not increase the risk	602 (49.1%)	204 (16.6%)	602 (49.1%)	420 (34.3%)
Pale pink/red urine can be a sign of kidney cancer	589 (48.0%)	589 (48.0%)	89 (7.3%)	548 (44.7%)
Lower back pain, lumps in the abdomen, and feeling tired frequently are signs of kidney cancer	548 (44.7%)	548 (44.7%)	70 (5.7%)	608 (49.6%)
The rise in obesity has been associated with a rapid rise in kidney cancer	510 (41.6%)	510 (41.6%)	126 (10.3%)	590 (48.1%)
There was no relationship between smoke and kidney cancer	450 (36.7%)	253 (20.6%)	450 (36.7%)	523 (42.7%)
High blood pressure is not associated with kidney cancer	438 (35.7%)	219 (17.9%)	438 (35.7%)	569 (46.4%)
Men are more likely to have kidney cancer	411 (33.5%)	411 (33.5%)	162 (13.2%)	653 (53.3%)
Urine test, blood test and physical examination will not detect early kidney cancer	252 (20.6%)	252 (20.6%)	412 (33.6%)	562 (45.8%)

Data are presented as *n* (%). “Correct rate” indicates the number and percentage of respondents who selected the correct answer (either “true” or “false” depending on the item).

### 3.4 Univariate and multivariate analyses of health literacy of four cancers

Univariate analyses showed that some independent variables, such as age, gender, smoking status, and alcohol consumption, were statistically significantly associated with the health literacy scores for the four cancers. The univariate analysis of health literacy of four cancers is presented in Supplementary Table 2. Significant independent variables (*p* < 0.05) of the four cancers were entered into a multiple linear regression model, respectively. The multiple regression analysis result is summarized in Table 5. Educational background and annual household income were significant influencing factors for the level of health literacy scores for all four cancers. Specifically, variables including age, gender, employment status, and family history of cancer influenced health literacy related to colorectal cancer. For esophageal cancer, individuals who achieved higher health literacy scores were older and had been diagnosed with cancer. Respondents who were older, female, retired, and had a family history of cancer demonstrated significantly higher health literacy scores for lung cancer. Moreover, in addition to educational background and annual household income, residents who reported a non-smoking status had higher health literacy scores for kidney cancer.

**Table 5:** Multivariable linear regression analysis of health literacy of four cancers (*n* = 1,226).

Dependent variable	Independent variables	Regression coefficient B**	Standard error	*β*	*t*	*P*	95% CI
Colorectal cancer^1^	Constant*	1.235	0.715	—	1.727	0.084	−0.168 ~ 2.639
	Age (years)	0.046	0.007	0.203	6.451	0.000	0.032 ~ 0.060
	Gender	0.899	0.208	0.134	4.316	0.000	0.491 ~ 1.308
	Educational background	0.726	0.109	0.196	6.669	0.000	0.512 ~ 0.939
	Employment status	−0.150	0.042	−0.100	−3.553	0.000	−0.233 ~ 0.067
	Annual household income	0.258	0.063	0.115	4.102	0.000	0.135 ~ 0.382
	Family history of cancer	0.745	0.233	0.089	3.204	0.001	0.289 ~ 1.202
Esophageal cancer^2^	Constant*	0.463	0.618	—	0.749	0.454	−0.750 ~ 1.676
	Age (years old)	0.035	0.007	0.166	5.232	0.000	0.022 ~ 0.048
	Educational background	0.715	0.104	0.205	6.902	0.000	0.511 ~ 0.918
	Annual household income	0.277	0.060	0.132	4.602	0.000	0.159 ~ 0.394
	Current diagnosis of cancer	1.806	0.509	0.099	3.545	0.000	0.806 ~ 2.805
Lung cancer^3^	Constant*	1.654	0.606	—	2.729	0.006	0.465 ~ 2.843
	Age (years old)	0.046	0.007	0.224	6.651	0.000	0.033 ~ 0.060
	Gender	0.619	0.175	0.100	3.536	0.000	0.275 ~ 0.962
	Educational background	0.536	0.100	0.158	5.346	0.000	0.339 ~ 0.732
	Employment status	−0.160	0.039	−0.117	−4.090	0.000	−0.237 ~ −0.083
	Annual household income	0.173	0.059	0.084	2.944	0.003	0.058 ~ 0.288
	Family history of cancer	0.612	0.218	0.079	2.808	0.005	0.184 ~ 1.040
Kidney cancer^4^	Constant*	2.162	0.497	—	4.346	0.000	1.186 ~ 3.138
	Educational background	0.556	0.098	0.174	5.672	0.000	0.364 ~ 0.748
	Annual household income	0.149	0.057	0.077	2.623	0.009	0.038 ~ 0.261
	Smoking status	−0.650	0.199	−0.094	−3.264	0.001	−1.040 ~ −0.259

*The constant value represents the non-random part of health literacy of cancer, which is not explained by the independent variables.

**Linear regression coefficient represents the number of units the dependent variable will change when the independent variable changes per unit, holding all other variables constant.

1: R^2^ = 0.126, the adjusted R^2^ = 0.120, F = 21.829, *p* < 0.001.

2: R^2^ = 0.104, the adjusted R^2^ = 0.098, F = 19.837, *p* < 0.001.

3: R^2^ = 0.095, the adjusted R^2^ = 0.089, F = 15.789, *p* < 0.001.

4: R^2^ = 0.056, the adjusted R^2^ = 0.052, F = 11.925, *p* < 0.001.

## 4 Discussion

Overall, our study showed that our samples of Chinese urban residents’ general health literacy of four cancers were moderate levels, with the highest scores for colorectal, followed by esophageal, lung, and kidney cancers. A possible explanation for this observation is that prevention strategies for colorectal and esophageal cancers are often targeted at modifiable factors such as diet and lifestyle. Due to the work of “Healthy China” public health movement and the strong championing efforts from the government, urban residents could easily access information related to improving modifiable factors (24, 25). In addition, the early symptoms of both cancers such as bloody stools/alternating diarrhea and constipation, or dysphagia are more obvious than those of lung cancer and kidney cancer, which prompts patients to seek medical help (3). It is recommended that efforts should be taken to improve public health literary regarding more “silent” cancers such as lung and kidney cancers, where there are less obvious symptoms that the public can recognize.

In addition, significant deficiencies were also identified, particularly concerning health literacy toward lung cancer. Although lung cancer is the most common malignancy and the leading cause of cancer death both globally and in China (26), the average correct rate of health literacy for lung cancer was only 58.8%. For instance, only half (50.7%) of the participants acknowledged that “lung cancer is the cancer with the highest death rate in China”. Furthermore, only approximately 43% of the residents recognized that lung cancer may be asymptomatic in its early stages and that females who never smoke could also develop lung cancer. The general population in China continues to hold the perception of “no smoking, no lung cancer”. In fact, the mortality of lung cancer among people who have never smoked is increasing (27). Studies also report that many people continue to have a fragmented knowledge about lung cancer (28, 29). Moreover, the study’s findings showed that there is room for improvement in residents’ knowledge and perception of lung cancer prevention, particularly regarding early symptom recognition and other risk factors that are not limited to smoking alone.

The markedly lower health literacy scores for kidney cancer warrant specific attention, which is consistent with the findings from other studies, which reported that the general population had low levels of perceived chronic kidney disease knowledge than other common chronic diseases (30). Several factors may explain this finding. First, kidney cancer receives substantially less public health promotion than lung and colorectal cancers in China. National cancer awareness campaigns, such as those under the “Healthy China” initiative, have historically prioritized more prevalent cancers, leaving kidney cancer with limited media coverage and community education programs (31). Second, early-stage kidney cancer is often asymptomatic—a characteristic reflected in our finding that only 20.6% of respondents knew how to detect kidney cancer early. This “silent” nature means individuals lack symptomatic triggers to seek information or medical consultation. Third, risk factors for kidney cancer—including hypertension, obesity, and specific genetic conditions such as von Hippel–Lindau syndrome—are less familiar to the general public compared to smoking-lung cancer associations (32). The low awareness of kidney cancer screening methods (only 49.9% recognized abdominal ultrasound as an early screening tool) suggests an urgent need for targeted educational interventions.

An interesting finding from our multivariable analysis was that retirement status was positively associated with lung cancer health literacy (with employment status coded such that retirement showed higher scores). This association may be explained by several mechanisms. First, retired individuals are typically older and thus face a higher absolute risk of lung cancer, which may increase personal relevance and motivate information-seeking behavior (20). Second, retirees have more discretionary time to engage with health information through media, community health lectures, and peer networks. Third, many community-based cancer screening programs in China specifically target older adults, providing structured opportunities for health education. Fourth, retired individuals may have greater cumulative exposure to health information over their lifespan. These findings suggest that workplace-based cancer education programs for working-age adults could help bridge the literacy gap between employed and retired populations.

While the association between socioeconomic status and health literacy has been well established, our study provides several novel insights. First, we demonstrate that education and income are consistently associated with health literacy across all four cancer types, suggesting that socioeconomic disparities in cancer literacy represent a generalized pattern rather than being cancer-specific. Second, the magnitude of these associations varied by cancer type, with the strongest effects observed for colorectal and esophageal cancers (*β* = 0.196 and 0.205, respectively) and the weakest for kidney cancer (*β* = 0.174). This gradient may reflect differential public exposure to cancer-specific health information. Third, our study extends previous findings to the Chinese urban context, providing empirical evidence to inform targeted interventions for lower socioeconomic status populations in China.

Consistent with previous studies (33, 34), our findings revealed that urban Chinese residents with higher educational background and higher annual household income report higher scores of health literacy regarding cancer prevention care, regardless of the types of cancer. Economic problems were usually suggested as a significant barrier to early screening of cancer. People in the lowest income level were less likely to undergo regular screening (35). This explanation is further supported by the fact that individuals with higher educational backgrounds and income may have better health literacy, which helps them more focus on healthy lifestyle (14). Moreover, these individuals may have access to better health resources or medical insurance; therefore, they have higher health literacy and a positive attitude toward cancer care (15). Based on these findings, it is recommended that efforts need to be made to develop targeted interventions, such as information that is accessible and appropriate for the lower-income and education population.

Implications for intervention development: Several interventions have been implemented to improve cancer health literacy in China, including the “Healthy China 2030” cancer awareness campaigns, community-based health lectures, and cancer screening promotion programs. However, our findings suggest that these efforts may not adequately reach lower education and lower income populations, nor do they sufficiently address kidney and lung cancers. Based on our results, we propose the following improvements: (1) Tailored materials: Develop cancer education materials at lower reading levels (e.g., pictorial and video-based) for populations with limited education; (2) Setting-specific delivery: Disseminate information through community health service centers and workplace wellness programs, not just online platforms, to reach lower-income individuals with limited Internet access; (3) Cancer-specific campaigns: Prioritize kidney and lung cancer awareness given their low literacy scores, emphasizing “silent” early symptoms and non-smoking-related risk factors; (4) Screening navigation: Provide financial navigation and logistical support for low-income individuals to reduce barriers to cancer screening.

## 5 Strengths and limitations

The strength of this survey is that it is based on a relatively large sample of urban residents living in different cities in mainland China, which demonstrate a portrait of Chinese residents’ health literacy of cancers. Additionally, this study compared the level of four cancer-related health literacy, providing a visible demonstration on healthcare providers and healthcare policymakers. Furthermore, this survey utilized a rigorously developed and tested cancer-related health literacy instrument, which can be used by other researchers to utilize and compare the results in different populations across various cultures.

However, this study has several limitations. First, due to the cross-sectional survey design and the recruitment method used, we could not establish the response rate. We also did not collect the geographical location of the respondents. This may inevitably bring selection bias, potentially limiting the generalizability of our findings to a broader population. Furthermore, we only investigate four major cancers, which calls for more types of cancers to be explored. In addition, we made all correct answers “true” in the survey. This design, while simplifying scoring, introduces a risk of acquiescence bias, where respondents may default to ‘True’ regardless of content. While the ‘Do not know’ option provided an alternative to guessing, the forced-response format required by the electronic platform to ensure data completeness may have inflated the scores. This is a common concern in knowledge-testing surveys where cognitive burden can influence response behavior. Future iterations of this instrument should consider balancing the proportion of ‘True’ and ‘False’ correct answers or employing multiple-choice items to better distinguish knowledge from response tendency, as seen in other recently developed cancer health literacy scales. Finally, the regression model utilized in this study yielded a relatively low adjusted *R* (2) value, indicating that other variables may need to be included to obtain more targeted results. Future studies should consider incorporating additional variables to enhance the predictive power of the model.

## 6 Conclusion

This cross-sectional survey conducted in China shows a relatively moderate level of health literacy of four major cancers. While education may contribute to the improvement in residents’ awareness, further investigation is needed to determine its impact on actual practice. The findings of this study indicated that higher educational background and higher annual household income were significantly associated with higher health literacy for all four cancers. Therefore, healthcare professionals should prioritize the development of intervention strategies aimed at the targeted population to better promote the effectiveness of the cancer screening and prevention programs that are accessible and appropriate for a diverse range of populations in China.

Future directions: The next steps for these findings include the following: (1) Intervention development and testing: Design and pilot culturally tailored, literacy-appropriate interventions targeting the identified high-risk groups (lower education and lower income) and low-literacy cancers (kidney and lung); (2) Rural population extension: Replicate this study in rural Chinese populations to compare urban–rural differences in cancer health literacy; (3) Longitudinal follow-up: Examine whether baseline health literacy predicts subsequent cancer screening uptake and early detection; (4) Scale refinement: Revise the cancer health literacy scale to include balanced response options and reverse-coded items to mitigate acquiescence bias; (5) Policy evaluation: Evaluate the effectiveness of existing cancer awareness campaigns in reaching socioeconomically disadvantaged populations.

## Data Availability

The raw data supporting the conclusions of this article will be made available from the corresponding author upon reasonable request, and if someone uses them for related researches.
