# Supplementary material for: Urban residents’ health literacy of four major cancers: a cross-sectional national survey
Source: Front Public Health. 2026 Jun 17;14:1762797. doi: 10.3389/fpubh.2026.1762797 (PMC13318984; doi:10.3389/fpubh.2026.1762797)
Supplement: Supplementary file 1 [file Table_1.DOCX]

**Supplementary Material Table 1. Demographic characteristics of respondents (n=1226)**

| **Characteristic** | **Mean±SD [Min,Max] / N (%)** | |
| --- | --- | --- |
| Age (years old) | 42.04 ± 14.84 [18,83] | |
| Living with relatives | 3.83 ± 1.46 [1,10] | |
| Gender | Male | 562（45.8%） |
|  | Female | 664（54.2%） |
| Marital status | Single | 249（20.3%） |
|  | Married | 913（74.5%） |
|  | Divorced | 55（4.5%） |
|  | Widowed | 9（0.7%） |
| Education background | Primary school or below | 32（2.6%） |
|  | Junior high school | 201（16.4%） |
|  | Senior high school | 309（25.2%） |
|  | Bachelor’s degree | 623（50.8%） |
|  | Master’s degree or above | 61（5.0%） |
| Employment status | Employed/working | 1014（82.7%） |
|  | Retired | 153（12.5%） |
|  | Student | 59（4.8%） |
| Annual household income (year) in 2024 | <20,000 RMB (<4,000 AUS $) | 180（14.7%） |
|  | 20,000-59,000 (4,000-11,800 AUS $) | 247（20.1%） |
|  | 60,000-149,000 (12,000-29,800 AUS $) | 350（28.5%） |
|  | 150,000-299,000 (30,000-59,800 AUS $) | 217（17.7%） |
|  | >300,000 (> 60,000 AUS $) | 97（7.9%） |
|  | Reject to answer/ Not sure | 135（11.0%） |
| Existing co-morbidities (including cancer) | No | 878(71.6%) |
|  | Yes | 348（28.4%） |
| Family history of cancer | No | 984（80.3%） |
|  | Yes | 242（19.7%） |
| Current diagnosis of cancer | No | 1189（97.0%） |
|  | Yes | 37（3.0%） |
| Smoking | No | 942（76.8%） |
|  | Yes | 284（23.2%） |
| Alcohol drinking | No | 814（66.4%） |
|  | Yes | 412（33.6%） |

**Supplementary Material Table 2. Univariate analysis of health literacy of four cancers (n=1226)**

| **Characteristic** | | **colorectal cancer** | | **esophageal cancer** | | **lung cancer** | | **kidney cancer** | |
| --- | --- | --- | --- | --- | --- | --- | --- | --- | --- |
|  |  | **mean ± SD** | **Statistics / *P*** | **mean ± SD** | **Statistics / *P*** | **mean ± SD** | **Statistics / *P*** | **mean ± SD** | **Statistics / *P*** |
| Age (years old) | | ***Pearson r*=0.142/0.000** | | ***Pearson r*=0.139/0.000** | | ***Pearson r*=0.150/0.000** | | ***Pearson r*=-0.062/0.031** | |
| Living with relatives | | *Pearson r*=0.009/0.746 | | *Pearson r*=-0.037/0.197 | | *Pearson r*=0.001/0.977 | | *Pearson r*=-0.052/0.069 | |
| Gender | Male | 6.98±3.731 | ***t’*=-4.932/0.000** | 6.07±3.296 | ***t’*=-2.053/0.040** | 5.55±3.202 | ***t’*=-3.42/0.001** | 4.40±2.979 | *t’*=1.086/0.278 |
|  | Female | 7.94±2.931 |  | 6.44±3.016 |  | 6.15±2.924 |  | 4.21±2.815 |  |
| Marital status | Single | 6.29±4.103 | ***H*=34.101/0.000** | 5.17±3.521 | ***H*=34.280/0.000** | 5.08±3.366 | ***H*=26.527/0.000** | 4.31±3.104 | *H*=0.231/0.972 |
|  | Married | 7.89±3.022 |  | 6.61±2.967 |  | 6.16±2.951 |  | 4.30±2.862 |  |
|  | Divorced | 6.82±3.328 |  | 5.98±2.991 |  | 5.07±2.788 |  | 4.17±2.341 |  |
|  | Widowed | 5.89±3.951 |  | 4.78±4.206 |  | 4.44±3.046 |  | 4.00±3.082 |  |
| Education background | Primary school or below | 5.09±3.888 | ***H*=45.375/0.000** | 4.28±2.505 | ***H*=50.111/0.000** | 4.53±2.973 | ***H*=39.860/0.000** | 3.93±2.434 | ***H*=52.868/0.000** |
|  | Junior high school | 6.56±3.598 |  | 5.23±3.237 |  | 5.20±3.159 |  | 3.35±2.801 |  |
|  | Senior high school | 7.07±3.658 |  | 6.13±3.325 |  | 5.41±3.066 |  | 3.93±2.760 |  |
|  | Bachelor’s degree | 8.10±2.913 |  | 6.73±2.932 |  | 6.39±2.905 |  | 4.66±2.919 |  |
|  | Master’s degree or above | 7.84±3.357 |  | 6.82±3.230 |  | 6.00±3.498 |  | 5.75±2.593 |  |
| Employment status | Employed/working | 8.38±2.425 | ***H*=35.641/0.000** | 6.36±2.692 | ***F*=3.637/0.001** | 6.45±2.539 | ***H*=75.084/0.000** | 3.73±2.614 | ***H*=31.827/0.000** |
|  | Retired | 8.64±2.046 |  | 6.80±3.094 |  | 7.33±2.544 |  | 5.08±2.835 |  |
|  | Student | 5.25±4.622 |  | 4.64±3.498 |  | 3.95±3.683 |  | 3.86±3.037 |  |
| Annual household income (year) in 2024 | <20,000 RMB (<4,000 AUS $) | 6.58±4.039 | ***H*=49.101/0.000** | 5.57±3.601 | ***H*=75.527/0.000** | 5.62±3.438 | ***H*=61.807/0.000** | 4.04±2.915 | ***F*=10.075/0.000** |
|  | 20,000-59,000 (4,000-11,800 AUS $) | 6.43±3.644 |  | 5.38±2.897 |  | 4.80±2.981 |  | 3.73±2.789 |  |
|  | 60,000-149,000 (12,000-29,800 AUS $) | 7.84±3.014 |  | 6.26±3.033 |  | 6.17±2.783 |  | 4.11±2.808 |  |
|  | 150,000-299,000 (30,000-59,800 AUS $) | 8.39±2.638 |  | 7.13±2.591 |  | 6.29±2.915 |  | 4.66±2.890 |  |
|  | >300,000 (> 60,000 AUS $) | 8.67±2.202 |  | 7.84±2.519 |  | 7.29±2.669 |  | 5.98±2.661 |  |
|  | Reject to answer/ Not sure | 7.50±3.532 |  | 6.37±3.676 |  | 5.78±3.283 |  | 4.34±2.945 |  |
| Existing co-morbidities (including cancer) | No | 7.44±3.424 | *t’*=-0.971/0.332 | 6.21±3.227 | *t*=-1.154/0.249 | 5.77±3.164 | ***t’*=-2.018/0.044** | 4.38±2.936 | *t’*=1.669/0.096 |
|  | Yes | 7.64±3.170 |  | 6.44±2.948 |  | 6.15±2.795 |  | 4.08±2.767 |  |
| Family history of cancer | No | 7.35±3.413 | ***t’*=-3.243/0.001** | 6.25±3.207 | *t’*=-0.568/0.571 | 5.77±3.131 | ***t’*=-2.547/0.011** | 4.38±2.893 | ***t*=2.009/0.045** |
|  | Yes | 8.08±3.043 |  | 6.37±2.917 |  | 6.30±2.770 |  | 3.96±2.873 |  |
| Current diagnosis of cancer | No | 7.49±3.358 | *t*=-0.277/0.782 | 6.23±3.155 | ***t*=-2.437/0.015** | 5.85±3.075 | *t*=-1.608/0.108 | 4.29±2.883 | *t*=-0.543/0.587 |
|  | Yes | 7.65±3.268 |  | 7.51±2.745 |  | 6.68±2.729 |  | 4.56±3.193 |  |
| Smoking | No | 7.67±3.221 | ***t’*=2.976/0.003** | 6.32±3.196 | *t’*=1.056/0.292 | 5.87±3.015 | *t*=-0.072/0.943 | 4.62±2.783 | ***t*=2.094/0.036** |
|  | Yes | 6.94±3.711 |  | 6.10±2.991 |  | 5.89±3.243 |  | 4.20±2.917 |  |
| Alcohol drinking | No | 7.62±3.222 | *t’*=1.762/0.078 | 6.36±3.153 | *t*=1.421/0.156 | 5.90±2.996 | *t’*=0.391/0.696 | 4.42±2.876 | *t*=1.063/0.288 |
|  | Yes | 7.25±3.591 |  | 6.09±3.141 |  | 5.83±3.209 |  | 4.23±2.899 |  |
